# Streamlining IRB review of AI human subjects research (AIHSR): the three-stage framework

**DOI:** 10.3389/fsysb.2026.1804193

**Published:** 2026-03-06

**Authors:** Tamiko Eto, Heather Miller, David Vidal, Mark Lifson

**Affiliations:** 1 TechInHSR, Madison, AL, United States; 2 North Star Review Board, Seattle, WA, United States; 3 Mayo Clinic, Rochester, MN, United States; 4 Mayo Clinic, Walla Walla, WA, United States

**Keywords:** ai governance, artificial intelligence, human research protections, IRB review, practical frameworks, research compliance, research ethics

## Abstract

Oversight of Artificial Intelligence in Human Subjects Research (AI HSR) presents unique challenges. These challenges arise from both the non-linear and iterative nature of AI development, as well as from the way AI shifts risk from individual research subjects to larger populations affected by AI-driven decisions and data handling. Traditional Institutional Review Boards (IRBs) often struggle to keep pace with these changes, which can lead to gaps in risk assessment and delays in the review process. There is a growing need for transparent, repeatable methods to manage AI risk in healthcare. This paper introduces the Three-Stage Framework, a risk-based oversight model designed to align ethical and regulatory review with an AI project’s stage of maturity and potential human impact. By aligning the level and timing of IRB review with the types of risks present at each stage of AI system development, the framework supports appropriate regulatory pathways and documents expectations while maintaining effective protection of human subjects. Through gradual, stage-appropriate documentation, the approach supports responsible and adaptive innovation while preparing AI systems for safe and ethical use across biomedical, social, behavioral, and educational domains. This approach prepares AI systems for safe and ethical use, accelerates compliant research, and helps IRBs and institutions maintain trustworthiness while protecting human subjects.

## Summary

This paper outlines the development of a streamlined and compliant review process for AI Human subjects Research (AI HSR). The proposed approach centers on the Three-Stage Framework, which applies a risk-based model to match oversight requirements with the maturity and risk level of AI projects across biomedicine, the social sciences, behavioral and educational research.

Many AI ethics frameworks have limited measurable impact on health outcomes (Porsdam Mann et al., 2025a; Chan et al., 2025). The Three-Stage Framework was originally informed by biomedical regulatory guidance, particularly the International Medical Device Regulatory Forum (IMDRF) 2017 guidance embeds established research ethics principles within this regulatory guidance. The framework applies universally to any context in which AI systems are developed using human data and have the potential to affect human participants. The Three-Stage Framework reflects the growing shift in AI ethics literature from articulating *what* ethical principles should govern AI to operationalizing *how* those principles can be implemented in real-world research and governance settings.

### Positioning the framework within existing literature

A growing body of literature addresses the intersection of AI and ethical research oversight. However, a review of the most relevant publications reveals that the existing literature centers around three common themes, none of which address the core problem that the Three-Stage Framework is designed to solve.

The first theme concerns the use of AI to streamline IRB processes. Godwin et al. (2024) developed a generative AI tool to auto-draft IRB applications. Porsdam Mann et al. (2025b) propose application-specific LLMs for pre-review screening of submissions. Hey et al. (2025) fine-tuned an open-source LLM to assist investigators in identifying ethical issues before IRB submission. These contributions focus on adopting technology to improve the efficiency of research ethics processes and oversight structures which would require a foundational understanding of *how* to efficiently and effectively *operationalize* these tools. However, they do not address how *human reviewers* can effectively evaluate the risks and benefits of AI studies.

The second theme addresses research ethics considerations for the machine learning (ML) development lifecycle. McCradden et al. (2022) propose a three-stage ethics pathway that involves exploratory data access, silent evaluation, and prospective clinical evaluation, mapping research ethics obligations to the phases of clinical ML translation. This provides useful conceptual framing for researchers and ethicists, but it is oriented toward the investigators’ perspective rather than toward the IRB reviewer evaluating a submitted protocol. The coincidence of the “three-stage” structure should not be confused with the present Framework. McCradden et al.’s stages track the development lifecycle, whereas the Three-Stage AI HSR Review Framework structures the review process itself.

The third theme involves the application of high-level ethical principles to AI research oversight. Bernstein et al. (2021) describe a societal-risk reflection process tied to research funded at Stanford University. While this Ethics and Society Review (ESR) model introduces a valuable deliberative step, it operates as a pre-funding gate focused on societal-level risks rather than on IRB-level human subjects protections, and its iterative review structure may present challenges for research administration efficiency and turnaround times at institutions without comparable resources.

Makridis et al. (2023) come closest to the problem space addressed by proposing supplemental AI-specific questions for IRB review within the Veterans Affairs (VA) system. This contribution represents an important early effort to augment existing IRB processes for AI research. However, several limitations warrant consideration: the supplemental questions are derived from high-level “trustworthy AI principles”, primarily principles introduced through the U.S. Executive Order (EO) 13960 which was authored under the Trump Administration in 2020. EO 13960 was later operationalized by the National Institute of Standards and Technology (NIST) AI Risk Management Framework (RMF) of 2023 after incorporating an enhanced AI Executive Order (EO 14110) under the Biden Administration in 2023. It is worth noting that EO 13960 of 2020 was issued solely for federal agencies, such as the VA, and remains in effect, whereas the NIST RMF has gone through several iterations over time. These iterations were due to two changes in Administration (Biden of 2020 and Trump of 2025). The latest Administration (Trump, 2025) issued new Executive Orders in January of 2025 after the Biden Administration’s EO 14110 to remove any practices that incorporate ethical considerations for bias and fairness, a critical component of IRB review. As a result, bias and fairness considerations were removed from the NIST RMF. Consequently, institutions that rely on the EO 13960 or latest version of NIST RMF may face challenges with the durability of frameworks anchored to specific policy instruments and meeting the ethical Principle of Justic application required of the IRB. For an IRB, they point to a “federal AI Safety Standard” to justify why a researcher has met this crucial ethical principle. Essentially, requiring an investigator to affirm adherence to such principles becomes a moving target. More substantively, the module does not provide reviewers with structured decision logic for evaluating investigator responses, determining regulatory classification, or assessing AI-specific risks under the Common Rule (45 CFR 46) or FDA frameworks (21 CFR 56/812).

The pilot study described by Makridis et al. reports on reviewer attitudes such as “ease of assessing” (an efficiency and process-oriented measure) but does not demonstrate whether the supplemental questions changed review outcomes or improved participant protections—a critical piece of claiming the pilot “demonstrates efficacy in risk mitigation.” The module relies on investigator self-attestation to principles like transparency and fairness but does not structurally adjust the level of review or oversight based on the project’s technical maturity, which could lead to both inefficient and ineffective oversight. This distinction is critical as risks to human subjects shift substantially as AI systems progress from training on retrospective secondary data to influencing real-world decisions. For example, the module asks investigators to describe the “transparency of the AI system,” but does not offer reviewers criteria for evaluating the adequacy of a given response. What constitutes sufficient transparency may vary considerably depending on the type of AI involved and will likely change as the tool is being developed. Furthermore, the module assumes the IRB has already determined whether an AI project triggers IRB oversight and does not provide guidance on classifying the appropriate level of review (exempt, expedited, or full board), making device determinations under 21 CFR 812, or assessing the AI-specific risks compared to anticipated benefits of the study. Again, all these aspects may change depending on the stage of development or type of AI tool being developed or evaluated.

These observations are not intended to diminish the contribution of previous work which represents a meaningful step toward integrating AI considerations into IRB review. Rather, they illustrate the gap between principle-based supplements and sustainable and operational guidance that IRBs require. The concept of supplemental AI-specific IRB review tools including reviewer checklists, and decision trees has been developed and nationally disseminated through practitioner channels. Specifically, the AI HSR IRB Reviewer Checklist and Exempt Determinations Decision Tree (Eto, 2021) were publicly released under a Creative Commons license in 2021, presented through the Public Responsibility in Medicine and Research’s (PRIM&R) in May 2022, and subsequently adopted at multiple institutions. The Three-Stage Framework builds on and significantly extends this prior practitioner-developed foundation by providing stage-specific review criteria, regulatory classification guidance, and structured risk assessment that scales with AI system’s maturity and proximity to human impact.

### Origins and evolution of the three-stage framework

The Three-Stage Framework was initially informed by guidance from the FDA, IMDRF, and ISO Standards (e.g., 42001) on the development of Device Software Functions (DSF) which remain unaffected by changes in the Administration. Further, the Three-Stage Framework is specifically designed to bridge the gap between abstract ethical principles and technical execution by embedding long-standing ethical (e.g., Belmont Report) and regulatory frameworks into the total product lifecycle. These guidelines distinguish between “clinical evaluation,” which involves evidence generation without patient impact, and “clinical investigation,” which involves prospective studies that affect patient care. This regulatory structure provided a useful starting point for thinking about staged AI oversight. Unlike the FAIR-AI Framework (Wells et al., 2025), which focuses on clinical utility and workflow integration, our approach addresses research specific issues.

As we worked with researchers across disciplines, we recognized that the core insight, matching oversight to proximity to human impact, applied universally not just to biomedical AI research. For example, a student affected by biased AI-generated course recommendations may face ethical concerns similar to those faced by a patient affected by biased diagnostic AI. Although the specific risks and regulations differ, both situations require systematic oversight to protect individuals from the potential harms of investigational AI.

### Background: why we need the three -staged approach- intended functionality and use boundaries

Emerging technologies continue to challenge the boundaries of traditional IRB oversight (BRANY, 2025), specifically when it comes to understanding what their role is in the study. Before any AI development begins, research teams must articulate what we call the “intended functionality and use boundaries” statement. In FDA-regulated contexts, this is called “Intended Use.” Outside the medical device regulatory framework, researchers may also refer to it as “System Specification,” or “Application Scope.” Regardless of terminology, the purpose is the same. This statement must answer four questions.

1. WHAT is the AI system designed to do? (specific function, not general capability).
2. FOR WHOM is it designed? (target population, not “everyone”).
3. In what SETTING will it operate? (specific context of use)
4. What are the use BOUNDARIES (what decisions can and cannot be made with outputs).

Without clear boundaries, IRBs cannot evaluate risk of harm to participants, potential for scope creep, automation bias, and over-reliance, institutional misuse, mission creep, or justice and fairness concerns. Here are a few examples to illustrate an effective “intended functionality and use boundaries” statement.

#### Biomedical example: AI for tumor margin detection

WHAT: A computer vision system that highlights potential tumor boundaries on CT/MRI scans.
FOR WHOM: Surgeons planning oncological resections for adult solid tumor patients.
SETTING: Pre-operative surgical planning in a medical center.
BOUNDARIES: Decision support only; tool function is designed so that surgeon is required to serve as decision authority; not for use in emergency settings, pediatric cases, or post-operative assessment.

#### Education example: AI for identifying at-risk students

WHAT: A predictive model generating risk scores for student academic struggle based on engagement metrics, assignment submission patterns, and historical data.
FOR WHOM: Academic advisors supporting undergraduate students in large introductory courses.
SETTING: Public university with documented equity gaps in STEM retention.
BOUNDARIES: Early alert tool only; advisors contact students to offer support; output NOT entered into official student records and cannot be used for enrollment decisions, grade appeals, disciplinary action, or mental health crisis response; students may decline outreach.

#### Social/behavioral example: AI for suicide risk prediction

WHAT: A machine learning model predicting elevated suicide ideation risk based on school records, survey responses, and behavioral indicators.
FOR WHOM: High school counselors serving students grades 9–12 in districts with elevated suicide rates.
SETTING: Secondary schools with existing mental health support infrastructure.
BOUNDARIES: Counselor decision aid only; triggers discreet outreach with explanation of what variables contributed to the risk score, no mandatory intervention (can be overridden); output NOT entered into official student records and cannot be used for disciplinary action, special education placement, or external reporting; human clinical judgment supersedes all AI outputs; continuous monitoring for bias across demographic groups required.

Notice how each statement specifies both what the AI does and does NOT do. These documented functional boundaries provide reviewable constraints that support appropriate human oversight, enable the end user to override AI-generated output when necessary, and inform decision-making in the best interests of the person affected by the tool.

Different types of AI require different levels of regulatory oversight, which must be informed by an understanding of the AI system’s intended functionality and its stage of development. IRBs must recognize the shift from early knowledge-based AI systems, where human users retained control, to modern AI systems, where, to benefit from the AI system as intended, circumstances often lead the user to rely on the AI-generated output, introducing new risks, particularly when proper guardrails are not in place to ensure safety, effectiveness, transparency, and explainability.

IRBs must carefully assess the type of AI being developed, the intended functionality of the end product, and the potential risks associated with both explicit and implicit objectives. While early AI systems posed relatively low risks due to their predictable and transparent outputs, modern AI systems, particularly those involving machine learning and deep learning, require a more nuanced risk assessment. Furthermore, as regulatory frameworks evolve, IRBs must remain vigilant in applying established and new guidelines to protect human subjects and ensure scientific integrity in the research and development of AI digital health tools. The Three-Stage Framework was designed to streamline this task.

### Quality improvement of the three-stage framework via iterative refinement through national collaboration

Since its initial development in 2024, the Three-Stage Framework (formerly referred to as the “Three-Phase Framework”) (Biden, 2023) has undergone validation through continuous feedback and refinement across diverse institutional settings. This evolution calls for expanded, multi-stage ethical review in AI and data science research (Petermann et al., 2022). A tool that began as a biomedical-focused approach informed by FDA and IMDRF guidance has evolved into a universally applicable framework through systematic engagement with research institutions, professional organizations, professional opinions, and feedback and federal oversight bodies nationwide.

Between October 2024 and November 2025, the framework was presented at more than 28 academic institutions, medical centers, and professional conferences. These presentations spanned all major U.S. geographic regions and included institutions ranging from Ivy League universities to smaller institutions, demonstrating the framework’s broad applicability across institutional types and contexts.

This extensive dissemination process served to gain feedback to improve the model. Each presentation generated detailed feedback from IRB professionals, researchers, institutional compliance officers, and federal regulators.

#### Common themes that emerged across institutions

1. The need for more precise articulation of stage transitions.
2. More explicit connections to existing regulatory frameworks beyond FDA guidance.
3. Streamline the Three -Stage framework for oversight of AI-enabled clinical trials.
4. Requests for guidance and adaptation for non-biomedical research studies via adapting the biomedical terminology to support social, behavioral, and educational research.
5. Guidance and training on AI literacy and the use and implementation of the Three-Stage Framework, as recommended by national and international bodies.

## Methodology used to incorporate feedback of the three-stage framework

Fictitious relatable case studies were presented to groups based on the types of research they review. Attendees were encouraged to think out loud on how they would approach the study. Specifically, they were encouraged to consider risk, the approval criteria, and connections to the Belmont Report. Notes were taken during the meeting on how to improve the presentation process and how to simplify the framework to offer greater utility to the HRPP/IRB community. Because of meetings with >20 institutions, the refinement decreased over time, and the Three-Staged Framework was finalized.

### Utility of the three-stage framework

Based on feedback, we know that the tool has undergone formal adoption and/or adaptation to internal processes by 21+ institutions, demonstrating real-world utility. For example, recent toolkits provide structured guidance for IRBs evaluating AI protocols in clinical research using the three-stage framework (MRCT Center & WCG, 2025). Notably, the University of Washington has operationalized our Three-Stage Framework in its institutional review process, providing a detailed rubric for applying the Belmont Principles to AI-specific risks (University of Washington Office of Research, 2025). Implementation teams also report overall improvement in reviewer confidence when using the framework’s structured approach. Feedback from the presentations stressed that the meeting attendees were empowered and more confident to review studies that include AI. For example, “We received a lot of positive feedback from attendees who were super grateful for the walkthrough. I’m happy to have been able to offer this to our members and cannot thank you enough for doing it!” and “We especially appreciated you sharing the three-phase framework for reviewing research involving AI. This was helpful it was in guiding our IRB members understanding of this complex and evolving area.”

### Additional tools created based on feedback

Ethical interventions are most effective when tailored to the specific stage of AI development (Hladikova et al., 2025). The AI Human Subjects Research (AI HSR) Risk Reference Tool, developed as a companion resource, has been refined through iterative testing at institutions that have provided ongoing feedback. The purpose of the tool is to help reviewers identify relevant risks for specific AI models/types and stages. The tool provides relevant risk-mitigation recommendations based on the identified risks.

The tool was created to further engage and empower IRB Reviewers to correctly identify AI risks and understand how they vary by AI stage and type. For example, LLMs pose fundamentally different risks than predictive risk models (Chan et al., 2025). The AI Human Subjects Research (AI HSR) Risk Reference tool helps prevent reviewers from treating a low-risk study as high-risk, and from treating high-risk AI tools as low-risk AI tools. This ensures that participants have adequate protections in place.

Through systematic presentations, feedback collection, iterative refinement, and documented implementation across diverse settings, the framework has demonstrated a practical, scalable approach to AI oversight that maintains scientific rigor across research domains.

### Importance and rationale of using the three-stage framework: institutional liability and building trustworthiness

Scientific evidence is the foundation of trust. Further, trust is foundational for data-driven health systems (Whicher et al., 2021). Historically, medical devices and decision-support tools were developed by external manufacturers and then brought to hospitals and schools for evaluation or adoption. In those cases, responsibility for design, validation, and regulatory compliance largely rested with the manufacturer.

This model is changing rapidly in the context of AI. Today, clinicians and educators increasingly use readily available AI tools or build their own systems for internal use or for research purposes. While well-intentioned, these individuals often have limited training in AI development, software validation, regulatory navigation, and risk management. As a result, the complexity, potential bias, and downstream risks of AI systems are frequently underestimated. Without appropriate methodologies and oversight, these systems may perform poorly, introduce bias, or cause harm, with the burden of that harm falling on both the individuals whose data were used during development and testing and the people on whom the tool will eventually be used.

As AI tools are developed within institutions, those organizations may effectively become the legal manufacturers of the resulting technologies. This shift in responsibility creates significant institutional risk that extends beyond the research and development setting. When AI systems move from research and testing into real-world use, these healthcare providers, researchers, and educators may become responsible not only for their own research participants but also for all individuals and the public affected by the system’s outputs.

For this reason, institutions must work closely with IRBs to ensure that risk management activities are applied consistently and at the appropriate times throughout all stages of AI development. The three-stage framework’s focus on gradually building documentation ensures that, by Stage 3, institutions have a strong evidence base to support informed deployment decisions or to determine that a system should not advance to production.

The approach provides a structured framework that articulates a process that, overall, considers the ethical impacts on individuals. This process helps mitigate possible downstream effects of AI in research by building and sustaining stakeholder trust. Finally, using the three-stage framework, conducted under IRB oversight and in coordination with the broader HRPP, protects not only the institution’s reputation, but most importantly, individual research participants now and in the future.

### An ethical risk-based approach to the IRB AI HSR review process

The Three-Stage Framework is designed to streamline ethical and regulatory review of AI research by matching oversight to the AI project’s maturity and risk. The framework follows a step-by-step progression.

Stage 1: Teaching and Training the System
The AI is learning from data but is not providing advice or making decisions.
Stage 2: Evaluating System Behavior
The AI produces outputs, but these outputs are tested and reviewed rather than used in real situations.
Stage 3: Real-World Use and Impact
The AI is used in real settings and its outputs influence decisions and outcomes. At this stage, the focus is on evaluating safety, effectiveness, and potential impact on people.

Rather than applying the same level of review to all projects or using partial approvals that can create oversight gaps, this framework provides a flexible, scalable pathway. It ensures strong oversight at the points where risk is highest, supporting responsible innovation while reducing the risk of regulatory non-compliance and building trustworthiness within the research evaluation process.

### Four guiding principles of the three- stage framework

The Three-stage Framework is guided by four principles to insure ethical oversight, trustworthiness, and the tool’s usability.

1. Risk-Based and Context Dependent:

The level of oversight scales up or down depending on the project’s stage and its potential to impact on people. Early-stage research with no direct human impact receives a review focused on data ethics. Later-stage research that affects real-world decisions receives a review focused on both indirect and direct impact.

2. Context Appropriate Language:

The framework applies across research fields, and the terminology is adapted to fit the research domain. For example, medical research may use the term “clinical evaluation” while education research may use “pedagogical validation.” Although the language changes, the ethical standards and alignment with regulatory expectations is the same.

3. Adaptive and Flexible:

AI research often develops in cycles, rather than in a straight line. This framework allows projects to move between stages as systems are refined, updated, or reevaluated, ensuring that oversight remains aligned with the system’s current state (Hladikova et al., 2025).

4. Gradual Documentation Generation:

Each stage builds on the previous one by adding new layers of documentation and validation. This step-by-step approach allows for the gradual, but necessary, collection of evidence that supports ethical, scientific, and regulatory accountability. By Stage 3, researchers have a complete record demonstrating the AI system was developed responsibly at every phase.

### Detailed stage progression of the three -stage framework

Stage 1: Discovery and Ideation

This is the learning stage where the researcher is essentially “teaching the system”. During this discovery stage, the AI is being built and trained to recognize patterns, generate outputs, or make predictions. Humans are not yet using it to make decisions that affect them. This stage focuses on establishing that the AI can learn something meaningful from data. This is done by selecting accurate, representative, and fit data for the intended purpose and target deployment population.

Stage 1 is scientifically rigorous work establishing foundational capabilities. The IRB reviews these aspects to ensure the algorithm is built on solid scientific design and methodology. Primary risks to “human data subjects” include data integrity, representation, and consent. While privacy protections are critical in this phase, IRBs need to recognize that privacy protections alone are insufficient in Stage 1, and they need to focus on scientific integrity and the presuppositions of the research (i.e., the scientific literature, study design, etc.). Further, traditional ethical principles, even if applied, are often inadequate for the complexities of AI research (Petermann et al., 2022). Common risks in Stage 1 are illustrated below.

In the clinical context, Stage 1 generally does not involve interactions or interventions with human subjects. As a result, early work in this stage should not influence research participants, patient care, or clinical decision-making. It also should not yet involve any analytical or clinical validation. This stage is narrowly focused on building and testing hypotheses through iterative algorithm building, usually using retrospective and secondary-use datasets (data that have already been collected for a different purpose). Stage 1 involves identifying a defined software function by establishing an association through iteratively testing retrospective data against a hypothesis.

In social, behavioral, and educational research contexts, Stage 1 similarly does not involve interactions or interventions with human participants. At this stage, the AI system is developed using retrospective or secondary-use data, such as historical student records, prior survey responses, learning management system logs, or archived behavioral data collected for non-research purposes. The AI is trained to identify patterns or associations (e.g., relationships between course engagement metrics and academic outcomes) without influencing current students, teachers, or institutional decisions.

Similar to the clinical example above, Stage 1 work in these domains should not affect educational placement, grading, disciplinary actions, or access to services. No AI-generated outputs are provided to participants or decision-makers, and no real-time recommendations are acted upon. The research is limited to hypothesis generation and preliminary model development, such as testing whether patterns in historical data predict defined outcomes. At this stage, the study does not involve validating effectiveness, implementing in live systems, or evaluating impact on individuals. Instead, it focuses on establishing a defined software function by iteratively testing retrospective data against a clearly articulated research question, with the IRB ensuring that data sources, assumptions, and methodological choices are scientifically sound and ethically justified.

When study teams apply to use data for discovery, they must clearly outline this Stage’s limitations and narrow focus and acknowledge that other (future) stages are premature for the proposed research. At the end of Stage 1, IRBs must carefully scrutinize researchers’ claims in both IRB protocols and publications. During and after Stage 1, researchers should not claim that an AI tool is useful in real-world settings. Stage 1 only shows that an AI can learn patterns from past data; it does not demonstrate that the system behaves correctly when used by real people or in real situations. Making claims about real-world impact at this stage goes beyond the evidence and may not be scientifically valid nor ethically responsible. For example, a cancer-detection AI trained on historical images may identify patterns. Still, it cannot be claimed to help clinicians diagnose patients until it is tested in realistic workflows (Stages 2 and 3). Similarly, an AI trained on past student data cannot be claimed to support advising or identify at-risk students without testing its effects on real users.

Ambitious and broad aims (such as deploying AI modules for real-world use where the output could influence real-time decision-making) can be claimed as future aims to be submitted later for IRB review and approval, provided the current research is identified as focusing solely on non-interventional discovery. For instance, a study aims to integrate clinical, genomic, and imaging biomarkers to improve outcomes in rectal cancer. However, the IRB application must specify that the current scope uses only retrospective data to develop algorithms. Once the algorithm has established a clinical association, the study team will submit an IRB modification to proceed to Stage 2.

Performing this work as part of Stage 1 provides the added benefit of an annual review, typical for IRB applications subject to 21 CFR 56, to continually reassess. Stage 1 plays a critical role in ensuring that early AI development practices uphold transparency, accountability, and respect for human data subjects, thereby sustaining the trust necessary for ethical and practical AI research across all later stages.

Stage 2: Analytic and Performance Validation

In Stage 2, the researchers are seeing if the AI behaves correctly. In other words, Stage 2 asks, “Does the AI behave as intended when real people interact with it?” This is when analytic and performance validation occur. The AI is not making or directly influencing decisions, however its outputs are being reviewed, rated, compared, or interpreted by human participants. This is where researchers (and the IRB) determine if the system is ready for real-world use and, to what extent, that real-world use requires additional specific controls. In other words, this is the moment the study team is preparing to present evidence to pass governance oversight away from the IRB and onto the institution that will deploy this tool into live production environments. The focus in Stage 2 shifts to validating the algorithm established in Stage 1. This involves evaluating preliminary safety and performance.

The IRB reassesses the risk-benefit ratio, carries over early-Stage 1 findings, and adds additional risk controls (as needed). Adjustments are made to address issues identified in Stage 1, ensuring the algorithm performs as expected.

In the clinical context, Stage 2 serves as a pilot project and is generally limited to offline validation or conducted in addition to (but not influencing) the standard of care. In other words, the technology is tested separately from live patient data and clinical environments such as electronic medical record (EMR) systems. This ensures that the algorithm does not impact patient care or incidentally contribute to current or future decision-making. Without this limitation, the study would be considered a clinical intervention (appropriate for Stage 3) (Food and Drug Administration, 2025). The primary risks in Stage 2 are illustrated as follows.

For SBER, Stage 2 similarly functions as a controlled pilot phase focused on evaluating whether the AI behaves as intended when real people interact with it. At this stage, the AI system’s outputs may be shown to educators, advisors, counselors, students, or researchers for review, rating, or comparison, but those outputs must not be used to make or influence real-world decisions. The purpose is to assess analytic performance, usability, and potential bias while ensuring that no participant experiences direct consequences from the AI’s outputs. For example, an AI system designed to predict which students may need additional academic support may be tested using historical or simulated student profiles. Advisors or educators might review the AI’s risk scores and provide feedback on whether the outputs are understandable, reasonable, and aligned with professional judgment. However, these outputs must not be used to change advising plans, academic placement, grading, disciplinary actions, or access to resources. The AI remains operationally separate from live student records and decision-making systems.

Similar to the clinical example above, the IRB reassesses the risk–benefit ratio in Stage 2, carries forward findings from Stage 1, and requires additional safeguards as needed. These may include controls to prevent AI outputs from being entered into official records, requirements for human review and documentation, and plans to evaluate performance and fairness across demographic groups. If AI outputs were allowed to influence real educational or behavioral decisions, the study would move beyond validation and would appropriately fall under Stage 3.

Similar to Stage 1, the IRB protocol and approval letter must articulate clear limitations to the research in Stage 2. The IRB approval letter should outline specific control measures that ensure the AI outputs cannot influence real-world decision-making. For example, even if the study aims to evaluate the AI’s accuracy on untrained images, a human must always confirm the AI’s outputs, and such outputs must not be entered into official human records, whether that be school records or EMR.

With the stakes higher, the IRB should conduct a more rigorous review. This may involve a more rigorous statistical assessment of model performance (accuracy, precision, recall) articulated in the study design, as well as a plan for systematic bias and fairness assessment across demographic groups.

Systematic bias and fairness assessments across demographic and contextual subgroups are a critical component of the Belmont Report’s Principle of Justice. Usability and interpretability evaluation with intended users is critical, as well as safety stress-testing with adversarial or ambiguous inputs.

For example, IRBs will need to make a formal device determination for clinical studies in Stage 2 (i.e., is it a “medical device” or “non-device”?). In SBER, there is no direct equivalent to a clinical “medical device determination.” However, there is a clear functional and ethical parallel. Instead of asking whether an AI system qualifies as a regulated device, the IRB must determine whether the system meaningfully influences decisions that affect individuals’ rights, opportunities, treatment, evaluation, or access to services. In Stage 2, AI outputs may be reviewed, rated, or interpreted by humans, but they must not be used to guide real-world decisions or be entered into official records such as grades, advising notes, or disciplinary files. If AI outputs begin to influence academic placement, behavioral interventions, or access to resources, the research moves beyond validation and into interventional use, requiring heightened oversight. This determination is grounded in 45 CFR 46s focus on risk, interaction, and the use of identifiable private information, NIH’s definition of “clinical research” and potentially triggers the Protection of Pupil Rights Amendment (PPRA) (National Institutes of Health, 2025; U.S. Department of Education, 2025). It is also ethically anchored in the Belmont Principle of Justice, which requires careful evaluation of bias and fairness across affected groups.

Functionally, this “decision-influence determination” serves the same purpose in SBER research as a medical device determination does in clinical research: identifying when investigational AI transitions from evaluation to impact and when stronger safeguards are needed to protect participants from harm.

By the end of Stage 2, the researchers will have comprehensive validation data with diverse populations, evidence of Good Manufacturing Practice (as applicable), a formal risk and mitigation assessment, design controls and version management systems, and usability and human factors testing reports for the IRB to review to progress on to Stage 3.

Stage 3: Real-World Deployment (Research)

Stage 3 is using the AI tool in real settings. This is the stage where AI outputs are used to influence or make real decisions. The system is no longer just being observed. It is now influencing behavior, opportunities, or outcomes. This stage requires the highest level of oversight because potential harms are direct and immediate. AI now affects people’s real lives. For example, teachers may act on AI feedback. School Counselors may respond to AI risk scores. Clinicians may incorporate AI recommendations into their care plans.

IRBs must consider the potential consequences for individuals, including end users who are expected to trust AI-generated outputs, as institutions increasingly act on them. In Stage 3, risks may arise from the use of AI in high-stakes decisions such as medical care, education, employment, or justice; from coercive or punitive actions triggered by AI, like mandatory interventions; and from mission creep, where AI is deployed beyond its originally approved scope. Other concerns include power imbalances between institutions and individuals, psychological harm or stigma resulting from AI-driven labeling. These effects deter people from seeking services due to surveillance, inequitable access to benefits or protections, and the risk of model drift over time if performance degrades without retraining.

In the clinical context, Stage 3 involves clinical investigations and interventional clinical validation. At this point, the software function transitions from algorithm development and clinical association activities to formal clinical investigation. The AI algorithm begins to operate in real-world settings, and while it may or may not interact directly with research participants, it can still have a significant impact on them.

Another example in the clinical context would be using an AI-enabled Brain-Computer Interface (BCI) software under evaluation, which may also involve an investigational surgical technique. Each research application must be evaluated independently. The IRB must carefully assess the risks and benefits as the AI transitions from Stage 1 through Stage 3, from research to potential clinical application.

For SBER, Stage 3 AI systems can shape how institutions treat people and how they experience education, social services, or behavioral interventions. For example, an AI system may be used to identify students at risk of academic failure and trigger mandatory tutoring, alter course placement, or prompt disciplinary monitoring. School counselors or advisors may rely on AI-generated risk scores to decide which students receive support services or interventions. In social or behavioral research settings, AI outputs may influence eligibility for programs, prioritize individuals for outreach, or guide case management decisions. In each case, the AI directly affects how people are evaluated, supported, or constrained within institutional systems. IRBs must consider not only the risks to research participants but also the risks to end users (e.g., teachers, counselors, etc.) who are expected to trust and act on AI outputs.

Similar to clinical research, each Stage 3 application must be evaluated independently, with the IRB carefully reassessing risks, benefits, safeguards, and accountability mechanisms as the AI moves from development and testing into real-world use.

All previous work culminates in validating the software’s functionality in real-world environments and across different populations to ensure fairness and accuracy. This step is also one way to reduce bias and improve reliability. Stage 3 often starts with small studies to evaluate preliminary safety and performance, evolving into more extensive studies to assess the AI system’s efficacy and potential adverse events. Stage 3 generally involves some form of interaction with either the research participant, patient, or end user and may involve an intervention.

During Stage 3, study teams and the institution’s HRPP must communicate and work closely with the study team to distinguish between standard procedures (or the standard of care, if applicable) and investigational procedures. In other words, the protocol must articulate what is being tested as an investigational tool or process and what is usually done. It would have been done if this study had never existed.

**FIGURE 1:**
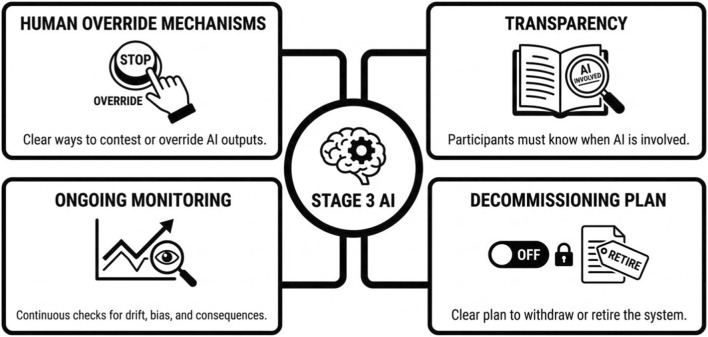
Critical Guardrails in Stage 3.

Once these distinctions are clearly articulated in the protocol, the IRB assumes a central role in safeguarding research participants by ensuring that appropriate oversight mechanisms are in place. Specifically, during Critical Guardrails in Stage 3 (Figure 1), the IRB must ensure the following critical guardrails.

1. Human Override Mechanisms: Participants and professionals must have clear, accessible ways to contest or override AI outputs without penalty.
2. Transparency: Participants should know when AI is involved in decisions affecting them, subject to appropriate exceptions (e.g., bias in research design). If deception is involved, a debriefing is mandatory.
3. Ongoing Monitoring: Stage 3 is not “set it and forget it.” For investigational AI systems, particularly those that are adaptive (not “locked”), continuously learning, or periodically updated, the IRB must ensure there is an ongoing monitoring plan to detect performance drift, emerging bias, and unintended harms. This monitoring plan should specify:
  - What performance, safety, and equity metrics will be tracked
  - How frequently monitoring will occur
  - Who is responsible for reviewing results and taking corrective action, and
  - Predefined thresholds or triggers for intervention, modification, suspension, or study termination.

For “locked” algorithms, monitoring focuses on real-world performance and unintended consequences. Any material change to model behavior, training data, or intended functionality must be evaluated to determine whether review or re-approval is required. These steps align with established regulatory and ethical principles, even if not explicitly called out, under 45 CFR 46.111(a) (1) and (a) (2), FDA SaMD and IMDRF principles, established AI risk literature, and IRB precedent for ongoing monitoring.

4. Decommissioning Plan: The IRB should ensure there is a clear decommissioning plan. This describes how an investigational AI system will be turned off, withdrawn, or safely retired when the study ends, is paused, or is stopped early. Should the appropriate governance bodies deem the tool to be safe to release into live production, outside of a research environment, where IRB protections are no longer in place, then those governance bodies take over all responsibility of the tool outside of the research context. This protects people, data, institutions, and ensures compliance.

At the end of Stage 3, the study team should have collected all the evidence needed to support real-world production outside of a protected research environment. This will include a complete quality management system, comprehensive user training materials, post-deployment surveillance and monitoring plans, incident response and adverse event reporting procedures, and regular performance reports with equity breakdowns.

### Building blocks: the three-stage framework

The Three-Stage Framework uses a “building blocks” metaphor. Instead of requiring all documentation at once (the current standard), it ensures a trustworthy final product by gradually adding layers of rigor and documentation, as appropriate, at each stage. This creates an audit-ready Design History File that demonstrates responsible development at every step. This approach serves organizational risk management, creates the documentation necessary for regulatory submissions, publications, or institutional accountability, and most importantly, promotes trust in protecting research participants.

## Conclusion

Through an interactive quality review process that gathers input from individuals, academic institutions, and independent professionals in the field, the Three-Phased approach has been refined into the Three-Staged Framework. The Three-Stage Framework aligns established research ethics and regulatory oversight with a project’s maturity. It offers a practical, flexible approach that can be used across many fields and research domains. Using this comprehensive framework can help accelerate innovation responsibly by leveraging a systematic review process that focuses on how AI is built and trained, and on potential risks, while maintaining the documentation needed. Finally, the Three Stage Approach helps ensure AI systems are safe, trustworthy, and ready for real-world use in any setting, promoting trustworthiness in the science and protecting human subjects.
